# Epicardial Fat Tissue Thickness in Preeclamptic and Normal Pregnancies

**DOI:** 10.5402/2012/389539

**Published:** 2012-10-15

**Authors:** Mehmet Mustafa Can, Esra Can, Olcay Ozveren, Ertugrul Okuyan, Burak Ayca, Mustafa Hakan Dinckal

**Affiliations:** ^1^Cardiology Department, Bagcılar Research and Education Hospital, Istanbul, Turkey; ^2^Gynecology and Obstetrics Department, Malatya Akcadag State Hospital, Istanbul, Turkey; ^3^Cardilogy Department, Yeditepe University, Istanbul, Turkey

## Abstract

*Background*. Epicardial fat tissue, another form of visceral adiposity, has been proposed as a new cardiometabolic risk factor, and the possible association of epicardial fat with hypertension has been shown in some recent studies. Although epicardial fat thickness (EFT) is associated with hypertension, the relationship between preeclampsia and EFT is still unknown. The purpose of this paper is to investigate the association between the echocardiographic EFT and the severity of preeclampsia in pregnant women. *Methods*. Forty women with preeclampsia were recruited and thirty-five normal pregnant women were matched for both maternal age and gestastional age served as control. The materials were collected immediately after delivery of the fetus, before placenta expulsion and before clamping of the umblical cord in patients and controls whom were in fasting state. Total cholesterol, high-density lipoprotein cholesterol (HDL), and triglyceride levels (TG) and low-density-lipoprotein cholesterol (LDL), and homeostatic model assessment of insulin resistance (HOMA-IR) levels were assessed. EFT was measured by using transthoracic echocardiography. *Results*. Among the preeclamptic women, 12 were diagnosed with severe preeclampsia and 28 mild preeclampsia. There were no statistically significant differences between patients with preeclampsia and normal pregnancy except when they are divided according to systolic and diastolic blood pressure, proteinuria levels, and parity and EFT levels. Among women with preeclampsia (*n* = 40), 30% had severe disease. Women with mild and severe preeclampsia had significiantly higher blood pressures at delivery and earlier gestational ages in comparison to control subjects. Although TG, VLDL and LDL, HDL, and HOMA-IR levels (*P* > 0.05) were comparable between preeclampsia and normal pregnancies, EFT levels were significiantly higher in patients with preeclampsia. Moreover, in subgroup analysis, patients with severe preeclampsia had higher EFT levels (*P* < 0.05) in comparison with mild preeclampsia. *Conclusions*. EFT levels measured at delivery were increased in patients with preeclampsia, and patients with increased levels of EFT levels had a substantially higher probability of the disease severity in comparison to those with mild preeclampsia and controls.

## 1. Introduction

Preeclampsia is the most important causes of maternal and neonatal mortality and morbidity in pregnancy [1]. Epicardial fat tissue, another form of visceral adiposity, has been proposed as a new cardiometabolic risk factor and the possible association of epicardial fat with hypertension has been shown in some recent studies [2, 3].

Although epicardial fat thickness (EFT) is associated with hypertension, the relationship between preeclampsia and EFT is still unknown. The purpose of this study is to investigate the association between the echocardiographic EFT and the severity of preeclampsia in pregnant women.

## 2. Methods

### 2.1. Subjects

The study has been approved by the Local Institutional Review Board and an informed written consent was obtained from each study participant before inclusion. In the current investigation, 40 women with preeclampsia were recruited and 35 normal pregnant women were matched for both maternal age and gestastional age served as control. Among the preeclamptic women, 12 were diagnosed with severe preeclampsia and 28 mild. Preeclampsia was diagnosed and classified according to strict criteria recommended by ACOG (2002): a systolic blood pressure of 140 mmHg or higher or a diastolic blood pressure of 90 mmHg or higher on two occasions at least 6 h apart occuring after 20 weeks of gestation in a pregnant woman with previously normal blood pressure and detectable urinary protein (>1+ by dipstick or 0.3 g/24 h and more) [4]. Clinical features of patients with severe preeclampsia (if any) were included as as a blood pressure greater than or equal to 160 mmHg/110 mmHg with either a urine dipstick showing 3+ or 4+ in a random urine sample or greater than 5 g of proteinuria over 24 h. Other evidence of severe disease included serum creatinine, eclampsia, pulmonary edema, oliguria (less than 500 mL/24 h), fetal growth restricition, oligohydramnios and symptoms suggesting significiant end-organ involvement (headache, visual disturbance). Women who met the citeria of preeclampsia but not severe preeclampsia were diagnosed as mild preeclampsia. Exclusion criteria were previous lipid metabolism disorders, history of dyslipidemia, multiple gestation, diabetes mellitus, chronic hypertension, infectious diseases diagnosed in pregnancy, premature rupture of membrane, active labor, polyhydramnios, kidney diseases, and signs of other concurrent medical complications. The control subjects had no sign of any gestational complication and fetal distress without evidence of hypertension or proteinuria and all gave health neonates of appropriate size for gestational age.

### 2.2. Sample Collection and Analysis

The materials were collected immediately after delivery of the fetus in patients and controls whom were in fasting state. Maternal venous was obtained in by puncturing the antecubital vein. Total cholesterol, HDL cholesterol, and triglyceride levels were measured on an autoanalyzer of the Konelab 60i analyzer (Konelab, Espoo' Finland). Low-density lipoprotein was calculated using Friedelwald's Formula [5]. Blood glucose was measured by routine laboratory techniques. Plasma concentration of fasting insulin was measured by using the commercial kit (BIOSOURCE, Camarillo, CA, USA). The homeostasis model assessment index for insulin resistance (HOMA-IR) was defined as fasting plasma insulin (mu/L) × fasting glucose (mg/dL)/405 by using Matthews's equation [6]. The coefficients of variation were less than 5% for every measurement.

### 2.3. Echocardiographic Assessments

Complete transthoracic two-dimensional echocardiograms were obtained from all patients. Standard parasternal and apical views were obtained in the left lateral decubitus position by using a Vivid 3 Pro ultrasound machine (GE Vingmed Ultrasound, Milwaukee, WI, USA). Images were digitally stored and reviewed by a single cardiologist blinded to the patients' information in order to avoid interreader variability. Two days after the first measurement, echocardiograms of 18 patients were randomly selected, and EFT was measured a second time to assess intraobserver variability. The left ventricular ejection fraction and the left ventricular (LV) and left atrial (LA) diameters were measured on M-mode traces recorded in the parasternal long axis view according to established standards [7]. Left ventricular mass (LVM) was calculated by using the Devereux Formula [8]. The epicardial fat was described as the echo-free space between the outer wall of the myocardium and the visceral layer of the pericardium. The epicardial fat thickness was measured perpendicularly from the free wall of the right ventricle at end-diastole in three cardiac cycles, as previously described [9]. The maximum EFT was measured from the point on the free wall of the right ventricle along the midline of the ultrasound beam perpendicular to the aortic annulus. For the midventricular parasternal short-axis assessment, maximum EFT was measured from the free wall of the right ventricle along the midline of the ultrasound beam, perpendicular to the interventricular septum at mid-chordal level and the tip of the papillary muscles, as the anatomic landmark. The intraobserver variability coefficient was calculated to be 0.72.

### 2.4. Statistical Analysis

Continuous variables are expressed as mean ± SD. The level of significance was 0.05. The Kolmogorov-Smirnov test was used for the normality test of all variables. To compare differences between patients with or without disease severity, Mann-Whitney *U* and Fisher's exact tests were used for continuous and categorical variables, as appropriate. Statistical analysis were performed using SPSS, version 15.0 for Windows.

## 3. Results

Baseline clinical and demographic characteristics of our study population are given in Tables 1 and 2. There were no statistically significant differences between patient with preeclampsia and normal pregnancy except when divided by according to systolic and diastolic blood pressure, proteinuria levels, and parity and EFT levels. Among women with preeclampsia (*n* = 40), 30% had severe disease. Women with mild and severe preeclampsia had significiantly higher blood pressures at delivery and earlier gestational ages in comparison to control subjects. Altough TG, VLDL, and LDL and HDL levels (*P* > 0.05) were comparable between preeclampsia and normal pregnancies, EFT levels were significiantly higher in patients with preeclampsia. Morover, in subgroup analysis, patients with severe preeclampsia had higher EFT levels (*P* < 0.05) in comparison with mild preeclampsia.

## 4. Discussion

The main findings of our study were that [1] EFT levels measured at delivery were increased in patients with preeclampsia, and [2] patients with increased levels of EFT had a substantially higher probability of the disease severity in comparison to those with mild preeclampsia or controls. To our knowledge this is the first study that evaluates the association between severity of preeclampsia with EFT. In recent studies although it was shown that many of the individual risk factors that compose metabolic syndrome are also risk factors in the development of preeclampsia [10]. These factors include visceral obesity, dyslipidemia, insulin resistance and blood pressure. Although our study group did not include any criteria of metabolic syndrome except higher blood pressure, patients with severe preeclampsia had increased EFT in comparison to those of controls and mild preeclampsia. This data provides insight into the relationship between EFT and preeclampsia. Epicardialadipose tissue is a type of visceral adipose tissue functioning as a metabolically active endocrine organ and the relationship between EFT presence and components of cardiovascular diseases have been described in several studies [11–14]. Moreover, it has been shown that epicardial adipose tissue is in direct contact with the myocardium, and it is very metabolically active and can secrete a large number of cytokines and vasoactive peptides, including free fatty acids, interleukin-6, TNF-*α*, angiotensin II, and plasminogen activator inhibitor-1 [15]. All of these molecules can independently increase the blood pressure. Although the causes of preeclampsia are completely unknown, one of the responsible mechanisms is thought to be activation of immune sytems with predominant involvement of cytokines and chemokines [16, 17]. Cytokines which are one of the important sources of inflamatory mediators have emerged as key cellular determinants of progression of the preeclampsia [18]. Previous articles alerted the scientific community that hyper activation of cytokines and adhesion molecules which were in concordance with the severity of diseaese may give prognostic data about the severity of the preeclampsia [19]. Recent studies have shown that cytokines which mediate the immune response to inflamatory disorders, were also secreted from epicardial adipocyte tissues. On the basis of these facts, EFT may be associated with the patholophysiological processes causing preeclampsia. There are also other several pathogenic mechanisms were thought to be the causative role for the association between increased EFT and the severity of the disease. Endothelial function plays a key role in determining the clinical manifestations of hypertensive disorders. Epicardial fat tissue seems to affect the endothelial function and increase sympathetic activity by its paracrine effect. Aydin et al. showed that EFT correlated with endothelial dysfunction assessed by flow-mediated dilatation in patients with metabolic syndrome [20]. Based on these data, associated endothelial dysfunction can be considered as one of the mechanisms of EFT for increasing blood pressure and severity of preeclampsia in pregnants. Although epicardial fat tissue is the true visceral fat depot of the heart, it is also an extremely active organ that produces cytokines and adipokines. Epicardial fat tissue-derived adiponectin production has been shown to be decreasing in patients with hypertension and decreased adiponectin has been shown to be related to increased collagen deposition and decreased arterial elastic properties [21, 22]. Moreover, recent studies reported that hypoadinopectinemia was associated with impaired endothelial dependent vasodilation and suggested that there is a link between epicardial fat tissue derived adinopectin levels and vascular tone [23]. Paolisso et al. showed that increased fatty acid concentrations may also stimulate the cardiac sympathetic system through an increase in plasma catecholamine concentrations [24]. We conducted this study in the experimental setting, and applied observational design, therefore, not drawing any definite pathophysiologic mechanisms for the association between increased EFT and the severity of preeclampsia. We speculate that the similar mechanisms may be attributable for increase in EFT level in PE and the cardiovascular disorders. Indeed, there is some data about possible association between epicardial fat and hypertension in literature. Several studies have shown a link between epicardial fat accumulation, and development of coronary heart disease and hypertension [3, 21]. Sironi et al. demonstrated that fat was preferentially accumulated intraabdominally and intrathoracically in newly found, untreated men with essential hypertension [25]. It was recently shown that early hypertension was associated with epicardial ectopic fat along with visceral fat [26]. Our study not only confirmed the results of these previous studies in hypertension with respect to increased EFT, may also expand the knowledge on the preeclampsia pathogenesis and assessment of its severity. There are a few limitations of the study worth mentioning. Although compatible in size with other similar investigations, this study was performed in a relatively small cohort, so chance represents a plausible alternative explanation. Many potentially important biomarkers and cytokines of preeclmapsia indices were not measured in the index study. We used a cross-sectional study design, and therefore, predictors of preeclampsia during follow period of pregnancy with EFT level measurements were not studied, and we included only patients whose EFT were normal before pregnancy. Finally, our study is too small, and observational design preclude us to adequately assess these important links. These data certainly should be confirmed in a larger, better randomized trial. In conclusion, maternal epicardial adipose tissue was increased in preeclamptic women, and increased EFT was associated with severity of preeclampsia.

## Figures and Tables

**Table 1:** Baseline characteristics of pregnant women with preeclampsia and those without preeclampsia.

Variables	Controls *n* (35)	Preeclampsia *n* (40)	*P* value
Maternal age	32 ± 3	34 ± 3	NS
Systolic BP (mmHg)	104 ± 8	154 ± 20	0.0001
Diastolic BP (mmHg)	72 ± 9	99 ± 8	0.0001
GA at delivery (weeks)	251 ± 20	235 ± 23	0.05
Protenuria	108 ± 32	1117 ± 667	0.0001
Total cholosterol (mg/dL)	167 ± 40	180 ± 49	NS
HDL cholosterol (mg/dL)	45 ± 13	45 ± 11	NS
LDL cholosterol (mg/dL)	98 ± 35	103 ± 42	NS
Triglyceride (mg/dL)	118 ± 52	147 ± 73	0.06
VLDL cholosterol (mg/dL)	23 ± 10	29 ± 6	0.06
FG (mg/dL)	93 ± 8	94 ± 8	NS
HOMA-IR	0.66 ± 0.2	0.72 ± 0.1	NS
LV end-diastolic diameter (cm)	5.2 ± 0.4	5.1 ± 0.3	NS
LV end-systolic diameter (cm)	3 ± 0.2	3 ± 0.3	NS
LA diameter (cm)	3.4 ± 0.2	3.5 ± 0.1	NS
EF (%)	66 ± 3	66 ± 4	NS
LVM (g/m^2^)	105 ± 11	104 ± 8	NS
EFT (mm)	5.6 ± 1.6	7.2 ± 2	0.05

Values are mean ± SD.

BP: blood pressure, GA: gestational age, y: year, *n*: number, HDL: high density lipoprotein, LDL: low density lipoprotein, VLDL: very low density lipoprotein, FG: fasting glucose, HOMA-IR: Homestatic model, assessment of insulin resistance, LV: left ventricular; LA: left atrium; EF: ejection fraction; LVM: left ventricular mass, EFT: epicardial fat thickness, NS: nonsignificant.

**Table 2:** Baseline characteristics of pregnant women with severe and mild preeclampsia.

Variables	Mild preeclampsia *n* (28)	Severe preeclampsia *n* (12)	*P* value
Maternal age	31 ± 3	33 ± 3	NS
Systolic BP (mmHg)	141 ± 7	167 ± 20	0.01
Diastolic BP (mmHg)	99 ± 7	99 ± 10	NS
GA at delivery (weeks)	245 ± 23	244 ± 19	NS
Protenuria	621 ± 127	1613 ± 639	0.0001
Total cholosterol (mg/dL)	166 ± 40	174 ± 46	NS
HDL cholosterol (mg/dL)	37 ± 7	38 ± 6	0.03
LDL cholosterol (mg/dL)	100 ± 32	109 ± 41	NS
Triglyceride (mg/dL)	142 ± 75	147 ± 86	NS
VLDL cholosterol (mg/dL)	28 ± 15	29 ± 17	NS
FG (mg/dL)	95 ± 8	96 ± 8	NS
HOMA-IR	0.6 ± 0.2	0.7 ± 0.1	NS
LV end-diastolic diameter (cm)	5 ± 0.2	5.1 ± 0.3	NS
LV end-systolic diameter (cm)	3 ± 0.1	2.9 ± 0.3	NS
LA diameter (cm)	3.4 ± 0.2	3.5 ± 0.1	NS
EF (%)	64 ± 4	66 ± 2	NS
LVM (g/m^2^)	102 ± 6	103 ± 7	NS
EFT (mm)	6.6 ± 0.6	7 ± 2	0.05

Values are mean ± SD.

BP: blood pressure, GA: gestational age, y: year, *n*: number, HDL: high density lipoprotein, LDL: low density lipoprotein, VLDL: very low density lipoprotein, FG: fasting glucose, HOMA-IR: Homestatic model, assessment of insulin resistance, LV: left ventricular; LA: left atrium; EF: ejection fraction; LVM: left ventricular mass, EFT: epicardial fat thickness, NS: nonsignificant.
